# Clinical and Laboratory Correlates of Cerebral Blood Flow Velocities and Risks for Stroke Among Steady-State Sickle Cell Anemia Children: A Cross-Sectional Study

**DOI:** 10.7759/cureus.92953

**Published:** 2025-09-22

**Authors:** Oyetoke C Oderanti, Samuel O Oninla, Moshood A Akintola, Olawale A Abayomi, Funso A Olagunju, Funmilola J Adesokan

**Affiliations:** 1 Department of Paediatrics and Child Health/Paediatric Hematology/Oncology, Osun State University Teaching Hospital, Osogbo, NGA; 2 Department of Paediatrics and Child Health/Paediatric Infectious Diseases/Paediatric Nutrition, Ladoke Akintola University of Technology, Ogbomoso, NGA; 3 Department of Paediatrics and Child Health/Paediatric Infectious Diseases/Paediatric Nutrition, Osun State University Teaching Hospital, Osogbo, NGA; 4 Department of Radiology/Paediatric Ultrasound, Osun State University Teaching Hospital, Osogbo, NGA; 5 Department of Paediatrics and Child Health/Paediatric Infectious Diseases/Paediatric Nutrition, Osun State University, Osogbo, NGA; 6 Department of Paediatrics and Child Health/Paediatric Neurology, Osun State University Teaching Hospital, Osogbo, NGA

**Keywords:** cerebral blood flow velocity, clinical and laboratory parameters, correlations, risks of stroke, sickle cell anemia in children

## Abstract

Background

Transcranial Doppler (TCD) ultrasound (US) is commonly used to assess cerebral blood flow velocity and identify abnormal flow patterns. Additionally, clinical and laboratory parameters could be useful for the early detection of sickle cell anemia (SCA) patients who are prone to developing stroke, especially in low-income settings.

Objectives

This study aims to correlate clinical and laboratory parameters with the risk of stroke according to TCD values in children with steady-state sickle cell anemia.

Methods

This study used a cross-sectional observational design, with study participants (steady-state SCA children, aged 2-14 years) recruited consecutively. The demographic details, general examination findings, frequency of crisis and blood transfusions, and duration of hydroxyurea usage, as well as the participants’ weight, height, and nutritional indices, were recorded. The time-average mean of maximum velocity (TAMMV) was determined via transcranial Doppler imaging US of the middle and anterior cerebral arteries (MCAs and ACAs) on both sides of the head. The full blood count was determined via a hematology autoanalyzer, and an enzyme-linked immunosorbent assay (ELISA) kit was used to measure fetal hemoglobin. Statistical Package for Social Sciences software for Windows version 26 (IBM SPSS Inc., Chicago, IL) was used to determine the frequency, mean and standard deviation, associations, and correlations. The level of statistical significance was set at P < 0.05.

Results

A total of 110 SCA children were studied. The TAMMVs for normal height and stunted subjects were 133.79 ± 27.32 and 154.74 ± 37.18 cm/s, respectively (t = 2.138, p = 0.035), and the prevalence of abnormal velocity was significantly greater among stunted subjects (c^2 ^= 6.25, p = 0.044). Age, weight, and height have significant negative correlations with TAMMV and p-values of 0.003, 0.009, and 0.002, respectively, whereas age and height have significant negative correlations with the risk of stroke (p-values of 0.048 and 0.046, respectively).

An increased WBC count was significantly associated with an increased risk of stroke (p = 0.000), whereas a decreased PCV significantly increased the risk of stroke (p = 0.009). WBC counts were correlated with the TAMMV (r = .227, p = 0.017) and risk of stroke (r = .390, p = 0.001), whereas PCV values were negatively correlated with the TAMMV (r = -.197, p = 0.039) and risk of stroke (r = -.286, p = 0.002). HbF levels were not associated with or correlated with the TAMMV or risk of stroke. The frequency of blood transfusion and crisis was not significantly associated with or correlated with TAMMV or the risk of stroke. Additionally, the duration of hydroxyurea usage was not significantly associated with TAMMV but was correlated with the risk of stroke (r = .195, p = 0.042).

Conclusions

Age, height, PCV, WBC count, and stunting status are significantly associated with and correlated with TAMMV and the risk of stroke. In children aged 2-5 years, stunted, with high WBC count and low PCV, TCD examination should be mandatory.

## Introduction

Sickle cell anemia (SCA) is a common genetic disorder that can be life-threatening [1]. Its life-threatening tendency stems from its severe complications, which include severe anemia, sequestration crisis, strokes, and infections. Among these complications, stroke is the most fearsome complication of sickle cell disease (SCD) [2]. Stroke is defined as a focal neurological deficit resulting from a neurological compromise that persists for more than 24 hours with evidence of a cerebral infarct corresponding to the focal deficit on neuroimaging assessment [3]. Stroke among SCA children is common, with a prevalence of 5%-7% in Nigeria [4,5], and it can be sudden with no warning signs [3]. It affects approximately 10% of patients with homozygous SCD, which is approximately 250 times greater than that in the general pediatric population [5,6]. SCA children are at high risk for stroke, and approximately 11% of them have at least one episode of stroke by 11 years of age [6], or up to 12% by the age of 21 years [7,8]. Some of the predictors of stroke in SCA patients are low fetal hemoglobin concentrations in the blood, abnormal blood flow in cerebral vessels, low hematocrit, and leucocytosis [9,10]. Early detection of SCA patients with a propensity to develop stroke is imperative to achieve good management outcomes [3,11].

In many previous studies, abnormal blood flow velocities in the cerebral arteries of SCA patients have been associated with increased risks of stroke [12,13]. Additionally, studies have shown that patients with higher levels of fetal hemoglobin tend to have normal TCD values and therefore a lower risk for stroke [12-14]. This finding was supported by Hokazono’s study in Sao Paulo, Brazil, which reported a mean HbF level of 18.5 ± 9.3% and a 1.6% high risk of stroke [15], whereas Nigerian studies reported HbF levels between 2.99% and 9.6%, with a high risk for stroke ranging from 4.6% to 10.8% [12,13,16]. However, Steinberg et al., on the other hand, reported no significant associations between HbF concentration and stroke, silent cerebral infarction, or sickle cell vasculopathy in SCD patients [17]. Hydroxyurea (HU) usage also affects cerebral artery velocity, and the use of HU progressively decreases the TCD value over time by increasing the packed cell volume (PCV) and HbF levels [12,18-20].

Stroke is a debilitating illness leading to prolonged morbidity and eventually mortality in SCA patients [11]. Its occurrence in a child with SCA destabilizes the family and increases the burden of care. Therefore, early detection of the risk of stroke in SCA patients via simple, inexpensive, and readily available clinico-laboratory parameters is needed, especially in resource-limited countries. In the literature, however, few studies [9,15] have correlated TCD values with clinical and laboratory parameters, especially in sub-Saharan regions. These studies only correlated clinico-laboratory parameters with TCD values and not with risks for stroke. Additionally, there is no clear identification of clinico-laboratory parameters that can be put together to guide the initiation of stroke preventive measures. Hence, there is a desire to know clinical and laboratory parameters that can predict the risk of stroke. Thus, this study focused on the relationship between risk of stroke and common clinical events, anthropometric measurements, and simple laboratory tests commonly performed in hospitals. The parameters of interest included the frequency of blood transfusions, frequency of crises, hydroxyurea usage, age, weight, height, PCV, WBC counts, and HbF levels to assess their relationship with risks of stroke. To this end, this study aimed to correlate the TCD values and the risks for stroke with the clinical and laboratory parameters of pediatric SCA patients to determine their associations.

## Materials and methods

Study design

This study is a prospective, cross-sectional, observational study among steady-state SCA children during routine clinic visits.

Study location and setting

The study was conducted at Ladoke Akintola University of Technology (LAUTECH) Teaching Hospital (LTH), Osogbo, Osun State, Nigeria (now Osun State University Teaching Hospital, Osogbo, Osun State, Nigeria). Steady-state SCA children accessing routine clinic at the pediatric hematologic clinic of the hospital were studied, with ultrasound (US) scans and hematology investigations carried out at the radiology and laboratory departments of the hospital.

Patient recruitment

Steady-state SCA children aged 2-14 years were recruited consecutively for the study, and the inclusion criteria were fulfilled.

Inclusion and Exclusion Criteria

Inclusion criteria: Steady-state children with SCA accessing the sickle cell routine clinic, aged 2-14 years, were included in the study. The steady state in SCA patients is defined as the period when the children with SCA are in an optimal state of health, free from all forms of crisis or other acute complications of SCA in the preceding month [12]. The subjects were assumed to be in a steady state when they satisfied the following criteria: (1) no fever at presentation and at least in the last four weeks, (2) no crisis at presentation and at least in the last four weeks, and (3) not on any medication apart from routine folic acid, vitamin c, proguanil, and those with or without hydroxyurea usage [12]. In addition, only children whose parents or guardians consented and subjects older than seven years assented were included in the study.

The exclusion criteria were children who had any crisis within the last four weeks before data collection, who were on chronic blood transfusion, or who had a stroke in the past. Additionally, children with HbSC or other hemoglobinopathies and children whose parents or guardians did not give consent, or children who were old enough and who refused assents, were excluded.

Sample size determination

The minimum sample size for this study was derived from the Leslie Kish formula: n = z^2^pq/d² [21], and the prevalence (6.9%) of abnormal high-risk blood flow velocity recorded by Oniyangi et al. [13] among patients with sickle cell disease (SCD). In the Leslie Kish formula [21], n represents the sample size calculated, P is the prevalence at 6.9%, q = 1-p, Z is the standard deviation at a 95% confidence level (1.96), and d is the level of precision (5%).

n = 1.96² × 0.069 × 0.931 / 0.05² = 98.7

Therefore, the minimum sample size for this study was 99 steady-state children with SCA.

Data collection

Before data collection, the study protocol was approved by the Ethical Committee of LTH, Osogbo, Nigeria, with the assigned approval number: LTH/EC/2017/04/308. Data for this study were collected between January and July 2018 (six months). The procedures (blood analyses and TCD ultrasound scans) involved in this study were explained to the parents/guardians and some of the study participants (old enough to understand). Thereafter, written informed consent was obtained from all the parents/guardians of the subjects and assent from some of the subjects (older than seven years). A semistructured questionnaire (proforma) culled from previous similar studies [9,15] was used to collect information from the participants. The information obtained included age, sex, genotype, frequency of crisis, frequency of blood transfusions, and usage of hydroxyurea. The data was cross-checked from the study participants’ hospital medical records and reconciled. Additionally, general examination findings, anthropometry measurements, laboratory results, and ultrasound scan assessment findings were recorded in the proforma.

Measurements

The weight and height of each of the study participants were obtained by standard methods. The raw weight and height values were converted into nutritional indices of height for age (H/A), weight for age (W/A), and body mass index (BMI).

During each transcranial Doppler imaging ultrasound velocimetry measurement at the radiology department, the procedure was explained again to both parents and the study participants. All the subjects’ TCD ultrasound scans were performed by a single radiologist, assisted by an ultrasound scanning-trained pediatrician. The study participants were reassured and remained calm, awake, and alert before and during the procedure. The middle and anterior cerebral arteries (MCA and ACA) of the circle of Willis were insonated on both sides of the head through the acoustic window of the temporal bone, which allowed direct visualization of the vessels. Color Doppler was activated with color-coded blood flow to identify the vessels (MCA and ACA). The time-average mean maximum velocity (TAMMV) of blood flow in both arteries (the MCA and ACA) was measured on both sides of the temporal bone and recorded. The procedure took 30 minutes to complete.

The mean velocity (TAMMV value) in centimeters per second (cm/s) for each artery was automatically determined by the TCD imaging ultrasound machine. The overall mean for each of the subjects was derived from the average blood flow velocities in MCA and ACA on both sides of the temporal bone.

Based on the Stroke Prevention Trial (STOP) criteria, where TAMMV values of <170 cm/s indicate standard risk, 170-199 cm/s indicate conditional risk, and ≥200 cm/s indicate high risk [22], each participant’s risk of stroke was assigned. As stroke can occur in any of the cerebral arteries, each participant’s risk of stroke was determined by the highest TAMMV value in any of the insonated arteries.

Hematologic parameters were determined via a Sysmex kx-21 N (hematology autoanalyzer) for full blood count and a CEA996Hu enzyme-linked immunosorbent assay (ELISA) kit by Cloud-Clone Corp, Wuhan, China, for fetal hemoglobin (HbF).

Statistical analysis

The data were analyzed via the Statistical Package for Social Sciences software for Windows, version 26 (IBM SPSS Inc., Chicago, IL). The data are presented in tables and figures. Continuous variables are expressed as the means and standard deviations. Means were compared via Student’s t‑test and analysis of covariance (ANOVA) where appropriate. Comparisons of categorical variables and tests for associations were performed via chi-square tests, and Pearson’s correlation analysis was performed. The level of statistical significance was set at P < 0.05.

## Results

A total of 110 steady-state SCA children were studied. Sixty-one (55.5%) participants were males, and 49 (44.5%) were females. The mean age of the study participants was 7.17 ±3.77 years.

Anthropometry

The mean height of the participants was 117 ± 21.97 cm (ranging from 80 to 166 cm), and their weights ranged between 10 and 59 kg, with a mean of 24.20 ± 11.53 kg. The mean and standard deviation (±SD) of H/A and W/A were 97.44 ± 6.55% and 97.38 ± 15.11%, respectively. BMI ranged between 10.60 and 26.30, with a mean of 16.67 ± 2.89. The mid-upper arm circumference of 48 subjects, aged 2-5 years, ranged between 14 and 18 cm, with a mean of 15.62 ± 1.06 cm.

Nutritional status in relation to TAMMV and risk of stroke

About 101 (91.8%) of the subjects had a normal height for age, and nine (8.2%) were stunted. The mean TAMMV value (SD) for the normal height subjects was 133.79 (27.32) cm/s, and it was 154.74 (37.18) cm/s for the stunted height subjects. The difference was statistically significant (t = 2.138, p = 0.035). Seventy-five (68.2%) subjects had normal BMI, 15 (13.6%) had underweight, and 20 (18.2%) were overweight. The mean TAMMV values ± SD of the subjects who had normal BMI (131.58 ±28.88), underweight (141.89 ± 33.62), and overweight (145.41 ±22.88) cm/s were compared, and the comparison yielded no significant differences (∆f = 2.337, p = 0.102). Table 1 shows the prevalence of the various types of risk of stroke in relation to nutritional status. A comparison of the distributions of the various types of risk of stroke between the subjects with a normal height for age and those with stunted height revealed significant differences (p = 0.044), with those with stunted height having a higher prevalence of high risk for stroke. Additionally, subjects who had an abnormal BMI (underweight and overweight) had a relatively greater prevalence of high risk for stroke; however, the difference was not statistically significant.

**Table 1:** Prevalence of various types of risks for stroke in relation to nutritional status Table 1 shows the prevalence of stroke risk in different nutritional statuses, and the chi-square test was used to determine the relationship. Poor nutritional status predisposes patients to the risk of stroke. Stunted children with SCA are more prone to have abnormal cerebral artery velocity, with a greater likelihood of stroke occurrence. SCA: Sickle cell anemia; H/A: Height for age.

Nutritional status	Risks for stroke, n (%)					
	Standard	Conditional	High	Total	c^2^	p-value
^♦^Norm H/A	53 (52.5)	35 (34.7)	13 (12.9)	101 (100)	6.252	0.044
Stunted	1 (11.1)	5 (55.6)	3 (33.3)	9 (100)		
Total	54 (49.1)	40 (36.4)	16 (14.5)	110 (100)		
Body mass index (BMI)						
±Normal weight	40 (53.3)	26 (34.7)	9 (12.0)	75 (100)	2.659	0.616
Underweight	7 (46.7)	5 (33.3)	3 (20.0)	15 (100)		
Overweight	7 (35.0)	9 (45.0)	4 (20.0)	20 (100)		
Total	54 (49.1)	40 (36.4)	16 (14.5)	110 (100)		

Age, anthropometry measurements, and nutritional indices correlated with TAMMV and risk of stroke

**Figure 1:**
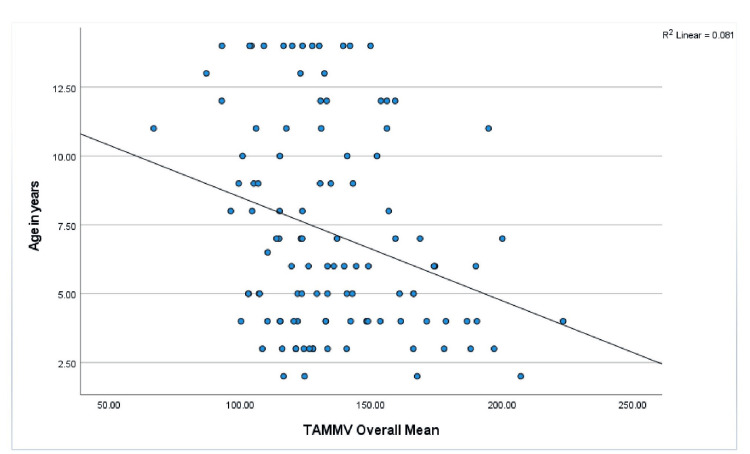
Correlation between TAMMV and age in years Figure 1 shows the effect of advancement in age on TAMMV. Pearson’s correlation analysis reveals that as children with SCA increase in age, the cerebral artery velocity decreases significantly. TAMMV: Time-average mean maximum velocity.

**Figure 2:**
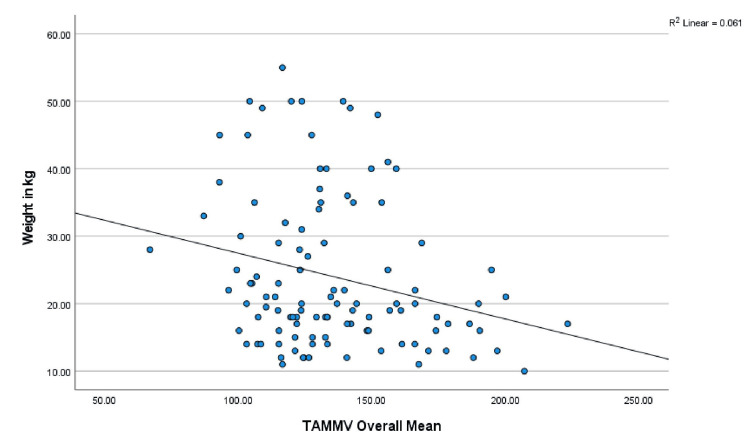
Correlation between the TAMMV and weight in kg Figure 2 depicts how weight affects cerebral artery velocity. Pearson’s correlation analysis shows that as children with SCA increase in weight, the cerebral artery velocity decreases significantly. TAMMV: Time-average mean maximum velocity; SCA: Sickle cell anemia.

**Figure 3:**
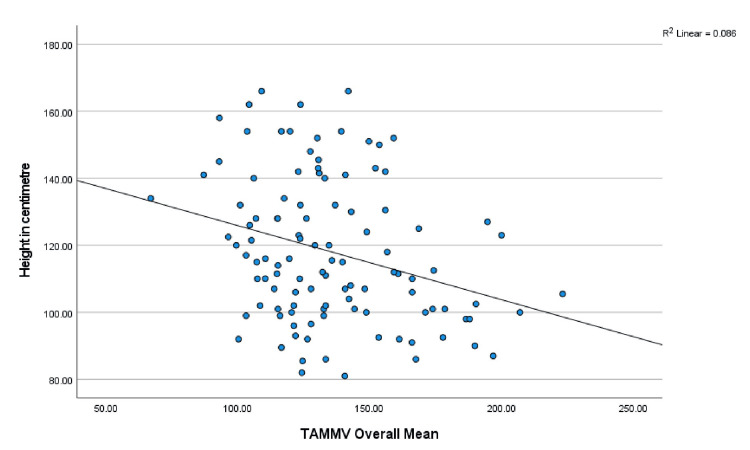
Correlation between the TAMMV and height in centimeters Figure 3 shows how the height of SCA children is related to the cerebral artery velocity. Pearson’s correlation analysis shows that as the height of children with SCA increases, the cerebral artery velocity decreases significantly. TAMMV: Time-average mean maximum velocity; SCA: Sickle cell anemia.

**Figure 4:**
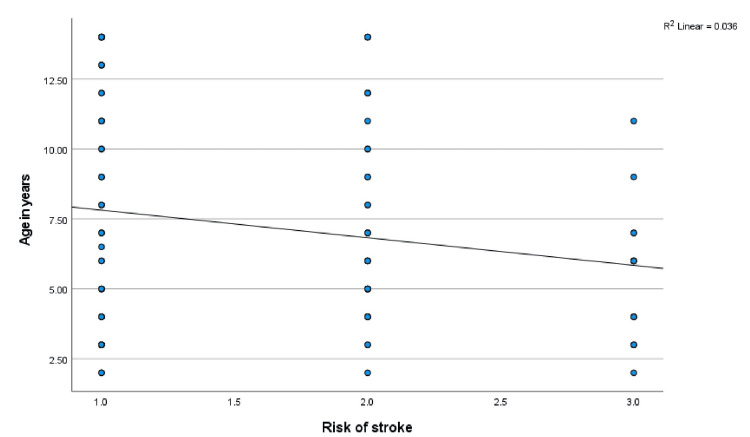
Correlation between the risk of stroke and age in years Figure 4 shows how age variations affect the risk of stroke. Pearson’s correlation analysis reveals that as age increases, the risk of stroke decreases, with the more severe form of risk of stroke significantly prevalent between five and six years of age.

**Figure 5:**
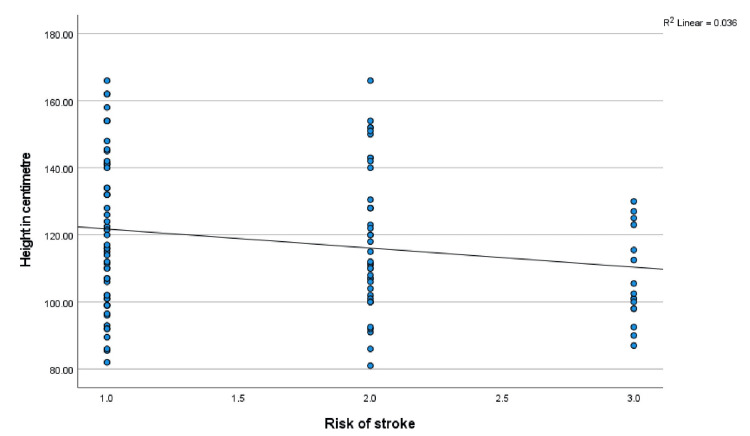
Correlation between the risk of stroke and height in centimeters Figure 5 shows the effect of height on the risk of stroke. Pearson’s correlation analysis reveals that as height increases, the risk of stroke decreases.

Age, anthropometry measurements, and nutritional indices were correlated with the mean TAMMV (age: r = -.285, p = 0.003; weight: r = -.247, p = 0.009; W/A: r = .034, p = 0.724; height; r = -.293, p = 0.002; H/A: -.105, p = 0.227; and BMI: r = .014, p = 0.887). Significant negative correlations were observed with age, weight, and height, and Figures 1-3 show scatterplots of the correlations. Additionally, the risks for stroke were correlated with age (r = -.189, p = 0.048), weight (r = -.166, p = 0.083), W/A (r = .034, p = 0.728), height (r = -.191, p = 0.046), and H/A (r = -.115, p = 0.230), and significant negative correlations were observed with age and height (Figures 4, 5 show the scatterplots).

Mean hematological parameters, TAMMV, and the risks for stroke

The means of the hematology parameters of the study participants with different stroke risks are shown in Table 2. The white blood cell count increased progressively and significantly as the risk for stroke increased in severity (p = 0.000). However, as the PCV decreased, the risk for stroke increased significantly in severity (p = 0.009).

**Table 2:** Mean heamatology parameters of the subjects and the risks of stroke Table 2 compares the mean of the hematology parameters of the subjects with different types of risk for stroke. Via one-way ANOVA, it was shown that there are significant differences between the mean WBC count and PCV of subjects with standard risk and subjects with high risk. Therefore, increasing WBC count and decreasing PCV value can predispose children with SCA to the severe risk of stroke. * indicates significant P-values. PCV: Packed cell volume; SCA: Sickle cell anemia; ANOVA: Analysis of variance.

Hematological parameters	Stroke risk types, n (%), Mean ± SD				
	Standard, 54 (49.1%)	Conditional, 40 (36.4%)	High, 16 (14.5%)	F-value	P-value
HbF (%)	4.46 ± 2.73	3.88 ± 2.67	5.03 ± 2.60	1.163	0.316
WBC (x 10^9 ^c/l)	8307.41 ± 3536.11	10446.50 ± 4246.66	13187.50 ± 5232.57	9.654	0.000*
PCV (%)	25.222 ± 2.97	24.23±3.82	22.31 ± 2.77	4.985	0.009*

Correlation of hematological parameters with TAMMV and risk of stroke

**Figure 6:**
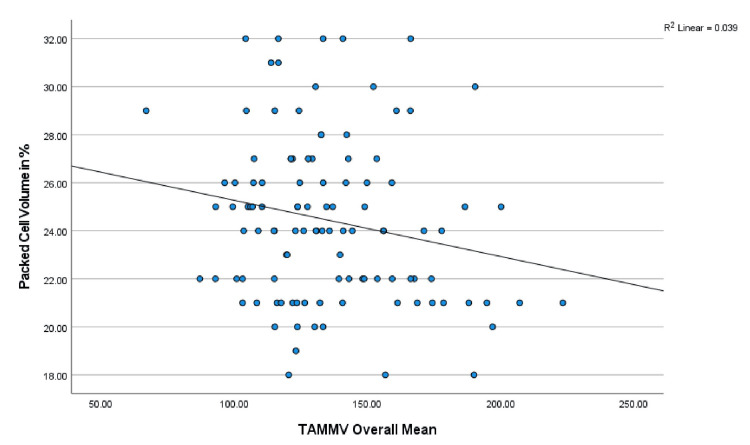
Correlation between TAMMV and PCV Figure 6 shows the effect of PCV levels on the cerebral artery velocity. Pearson’s correlation analysis reveals that as packed cell volume decreases, cerebral artery velocity increases (negative correlation). Thus, decreasing PCV increases the risk of stroke. TAMMV: Time-average mean maximum velocity; PCV: Packed cell volume.

**Figure 7:**
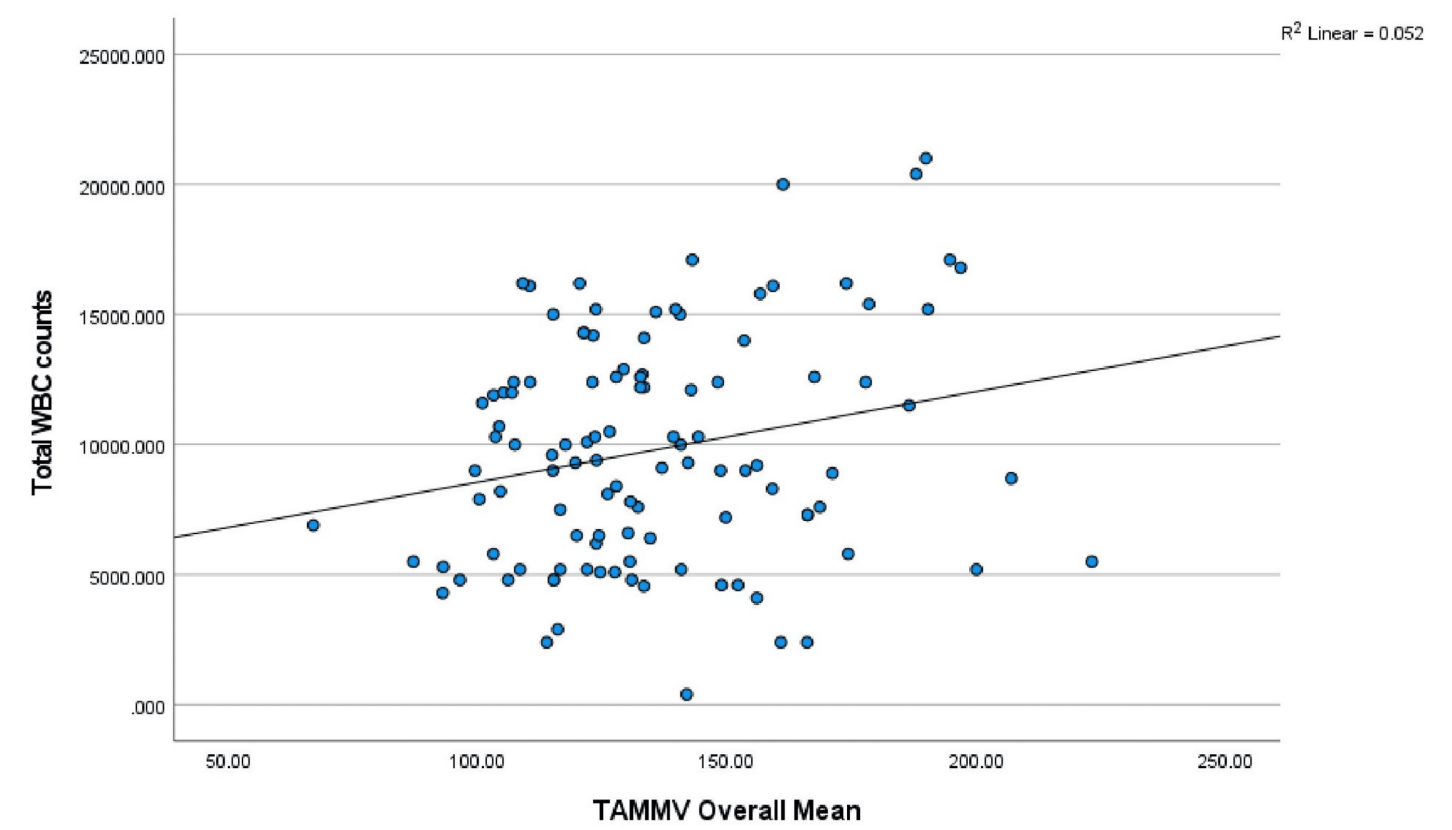
Correlation between TAMMV and WBC count Figure 7 displays the effect of WBC counts on the cerebral artery velocity. Pearson’s correlation analysis reveals that as WBC count increases, cerebral artery velocity increases (positive correlation). Thus, an increasing WBC count increases the risk of stroke. TAMMV: Time-average mean maximum velocity.

**Figure 8:**
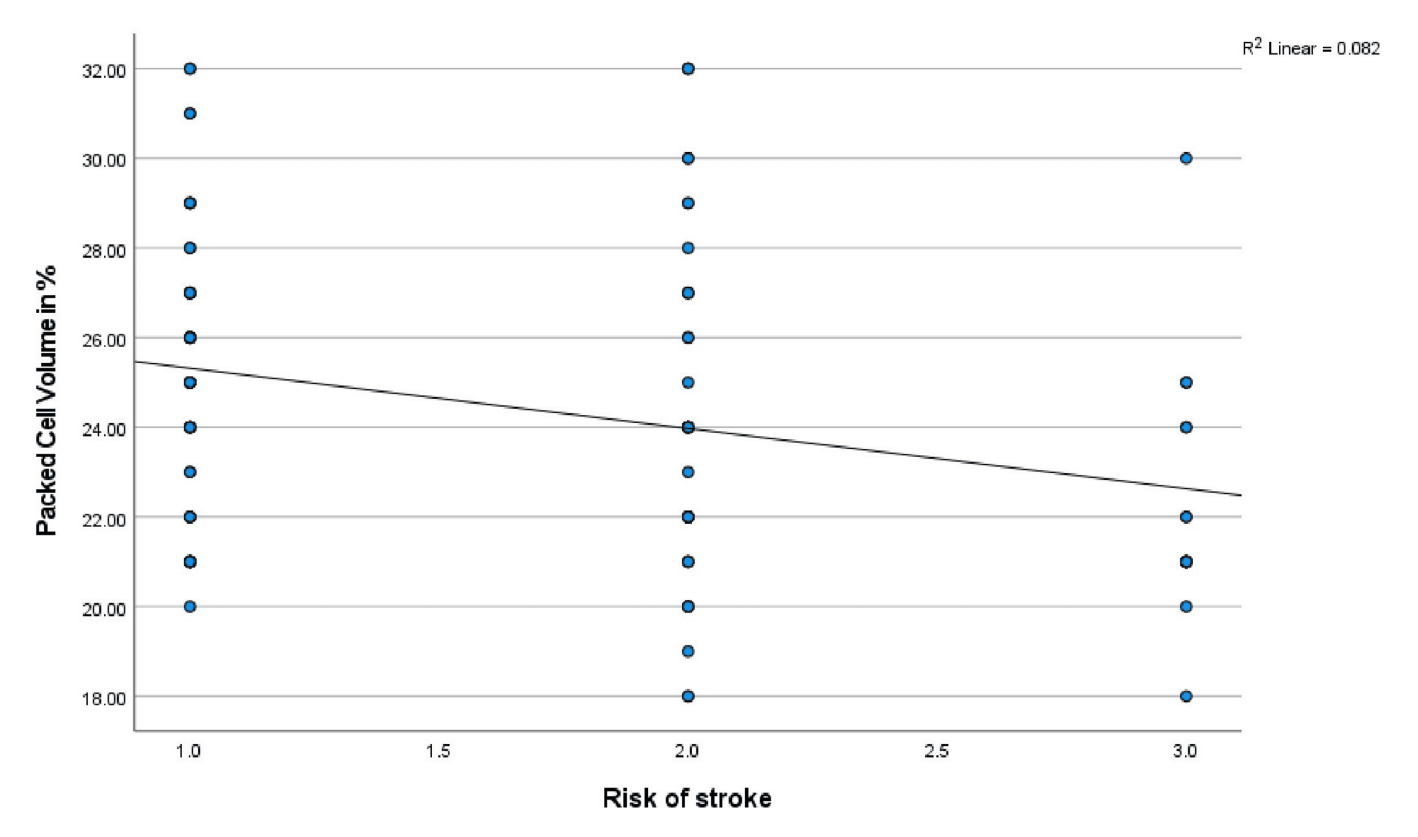
Correlation between the risk of stroke and PCV The effect of variations in the PCV on the risk of stroke is shown in Figure 8. Pearson’s correlation analysis reveals that a decrease in the PCV value progressively worsens the risk of stroke. PCV: Packed cell volume.

**Figure 9:**
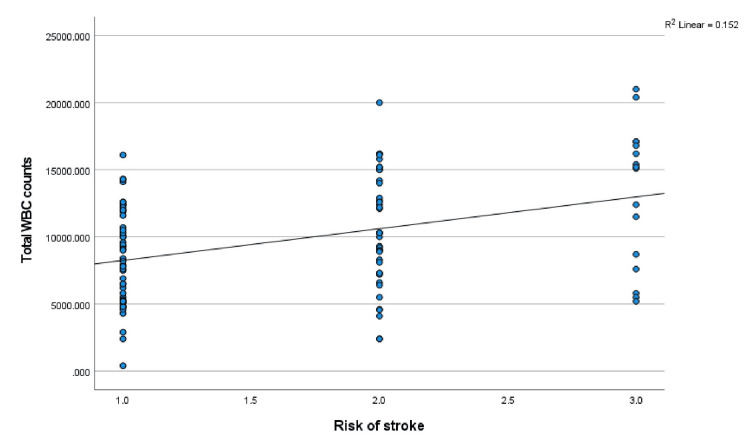
Correlation between risks of stroke and WBC The effect of WBC counts on the risk of stroke is displayed in Figure 9. Pearson’s correlation analysis reveals a positive correlation between WBC count and risk of stroke. Thus, an increasing WBC count progressively increases the risk of stroke.

Furthermore, the mean TAMMV values were correlated with hematology parameters (PCV, WBC, and HbF) via Pearson’s correlation analysis: PCV: r = -.197, p = 0.039; WBC: r = .227, p = 0.017; and HbF: r = .032, p = 0.737. A significant correlation was established between the TAMMV and both the PCV and WBC. The scatterplots are shown in Figures 6, 7, respectively. Additionally, the risk of stroke was correlated with the following hematology parameters: PCV: r = -.286, p = 0.002; WBC: r = .390, p = 0.001; and HbF: r = .020, p = 0.833. PCV and WBC count were significantly correlated with stroke risk (see Figures 8, 9).

Relating the mean TAMMV values and risks for stroke with hydroxyurea usage, frequency of blood transfusions, and crises

In Table 3, the mean values of TAMMV related to the duration of hydroxyurea use, blood transfusion frequency, and number of crises were compared. No significant differences were found. Furthermore, Table 4 shows the prevalence of various risks for stroke in relation to hydroxyurea use duration, frequency of blood transfusion, and number of crises, with no statistically significant differences observed.

**Table 3:** The mean values of TAMMV in respect of the duration of hydroxyurea usage, frequency of blood transfusion, and crises Table 3 shows the effects of several clinical parameters (hydroxyurea use, frequency of blood transfusion, and crises) on cerebral artery velocity. Using one-way ANOVA, the mean TAMMV of subjects with different periods of hydroxyurea use, numbers of blood transfusions, and crises were compared. There were no statistically significant differences in the mean. Thus, the clinical parameters do not affect cerebral artery velocity. TAMMV: Time-average mean of maximum velocity; ANOVA: Analysis of variance.

Duration and frequency (n)	TAMMV Values, Mean ± SD
*Duration of hydroxyurea usage*	
None (94)	134.10 ± 27.61
<6 months (2)	138.38 ± 21.62
≥6 months (14)	144.52 ± 35.74
F-value	0.815
p-value	0.445
*Frequency of blood transfusion within the last year*	
Nil (89)	134.58 ± 28.92
1-2 times (17)	141.24 ± 29.94
3-5 times (3)	134.13 ± 17.12
≥6 times (1)	123
F-value	0.311
p-value	0.817
*Frequency of crises within the last year*	
Nil (16)	147.90 ± 31.74
1-2 (Mild; 46)	133.57 ± 28.61
3-5 (Moderate; 38)	134.34 ± 28.64
≥6 (Severe; 10)	128.98 ± 20.83
F-value	1.274
p-value	0.287

**Table 4:** Prevalence of various types of risks for stroke in relation to the duration of hydroxyurea usage, frequency of blood transfusion and crises Table 4 shows the prevalence of various types of risk of stroke among the subjects with different periods of hydroxyurea use, numbers of blood transfusions, and crises. Via chi-square test, the prevalence of various types of risk for stroke among the subjects with different periods of hydroxyurea use, numbers of blood transfusions, and crises was compared. No significant difference was observed. Thus, the clinical parameters do not affect the risk of stroke.

Duration and frequency	Risks for stroke, n (%)					
	Standard	Conditional	High	Total	c^2^	p-value
Duration of hydroxyurea use						
None (94)	50 (53.2)	32 (34.0)	12 (12.8)	94 (100)	7.422	0.115
<6 mon^♦^ (2)	0 (0)	2 (100)	0 (0)	2 (100)		
≥6 mon^♦^ (14)	4 (28.6)	6 (42.9)	4 (28.6)	14 (100)		
Total	54 (49.1)	40 (36.4)	16 (14.5)	110 (100)		
Frequency of blood transfusion in the preceding 12 months						
None	48 (53.9)	30 (33.7)	11 (12.4)	89 (100)	11.209	0.082
1-2 times	5 (29.4)	7 (41.2)	5 (29.4)	17 (100)		
3-5 times	0 (0)	3 (100)	0 (0)	3 (100)		
6 or more	1 (100)	0 (0)	0 (0)	1 (100)		
Total	54 (49.1)	40 (36.4)	16 (14.5)	110 (100)		
Frequency of crises in the preceding 12 months						
None	6 (37.5)	7 (43.80)	3 (18.8)	16 (100)	1.441	0.963
1-2 crises	24 (52.2)	16 (34.8)	6 (13.0)	46 (100)		
3-5 crises	19 (50.0)	14 (36.8)	5 (13.2)	38 (100)		
6 or more	5 (50.0)	3 (30.0)	2 (20.0)	10 (100)		
Total	54 (49.1)	40 (36.4)	16 (14.5)	110 (100)		

Clinical parameters correlated with the TAMMV and the risk of stroke

**Figure 10:**
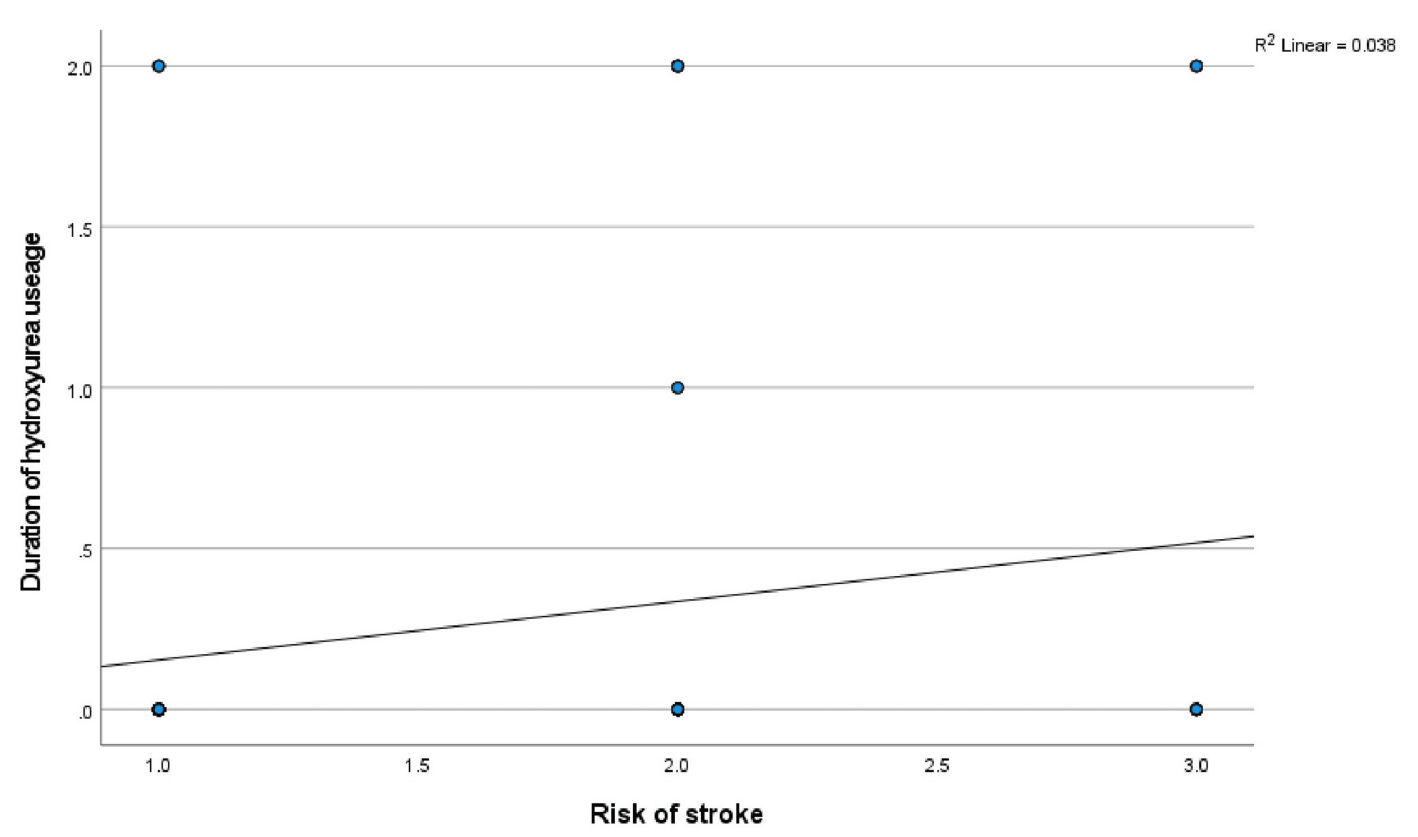
Correlation between risks of stroke and duration of hydroxyurea use Figure 10 shows the relationship between the risk of stroke and hydroxyurea use in this study. Pearson’s correlation analysis reveals that as the risk of stroke increases, the use of hydroxyurea increases (positive correlation).

The mean TAMMV values were correlated with the frequency of blood transfusions, number of crises, number of clinic visits in the last 12 months, and duration of hydroxyurea usage. The correlation coefficient and p values are as follows: frequency of blood transfusion: r = -.005, p = 0.956; number of crises: r = -.116, p = 0.229; clinic visits: r = -.061, p = 0.524; and duration of hydroxyurea usage: r = .122, p = 0.203. No significant correlation was observed. Additionally, the risk of stroke was correlated with the frequency of blood transfusion: r = .017, p = 0.862; the number of crises: r = -.062, p = 0.520; clinic attendance: r = -.022, p = 0.823; and duration of hydroxyurea usage: r = .195, p = 0.042. The risk of stroke was significantly correlated with the duration of hydroxyurea use (see Figure 10).

## Discussion

Nutrition is a natural need for all living things, and any lack or overindulgence could predispose an individual to disease conditions. Chronic malnutrition is detrimental to the health of children, especially those suffering from chronic diseases such as SCA. The significantly greater blood flow velocity (TAMMV) and greater risk of stroke among the stunted steady-state SCA children in this study attested to the adverse effects of poor nutrition. This finding is not surprising, as the nonavailability of adequate nutrients places additional stress on the body systems of SCA patients. This finding is similar to that of a study in Jamaica, which reported that increased TAMMV was correlated with height (p = 0.007) but not with height for age or body mass index (BMI) z scores [23]. In contrast, a Northern Nigerian study revealed that severe malnutrition in children with SCA was associated with a lower prevalence of abnormal TCD measurements [24]. The propensity of chronically malnourished subjects to record higher TCD values and a higher prevalence of abnormal risk for stroke in this study could be explained by the fact that malnutrition can exacerbate the severity of SCA complications, such as pain crises, acute chest syndrome, and stroke [25]. This is made possible through the presence of chronic inflammation and oxidative stress, factors that play roles in the development of chronic vasculopathy [26], i.e., stroke predisposing factors.

In this study, age was significantly negatively correlated with the risk of stroke. This finding is similar to the findings of earlier studies showing that children between two and five years of age have a high risk of abnormal TAMMV values and a high risk of stroke [5,9]. The risk is lowest before the age of two years, probably because of the protective influence of fetal hemoglobin on sickling [5,6]. It has been reported that TAMMV values decline progressively with advancing age [9,27], and with this, it was recommended that SCD patients in the first five years of life be priority targets for routine TCD examinations in settings of limited resources [9].

PCV and WBC count are closely related to the tendency of SCA patients to develop stroke. This could be explained by the fact that a low PCV puts patients in hyperdynamic circulation to meet the metabolic (oxygen) needs of the body. In this state, there is an increase in the force of heart contraction and heart rate, thereby leading to an increase in blood flow velocity in the cerebral vessels [10,28]. Low PCV has been reported to predispose SCA patients to stroke occurrence [12,29], and a study reported low hematocrit levels in more than two-thirds of patients with stroke [10]. WBCs, inflammatory cells that promote blood cell adhesion to the vascular endothelium, cause narrowing of the vascular lumen, which increases blood flow velocity. Therefore, the greater the number of WBCs, the greater the blood velocity. George et al. also noted a higher steady-state leucocyte count in more than three-quarters of patients with stroke [10]. This study was unable to establish significant associations or correlations between HbF and the risk of stroke. However, compared with the HbF levels reported in similar studies [12-16], the low mean HbF level (4%) and the high risk of stroke (14.5%) in this study support the fact that a low HbF concentration predisposes SCA patients to the risk of stroke. The failure to demonstrate associations or correlations between HbF and the risk of stroke in this study could stem from the low level of HbF in the present study participants. On the other hand, this study’s findings support Steinberg et al., who reported no significant associations between HbF concentration and stroke, silent cerebral infarction, or sickle cell vasculopathy in SCD patients [17]

The frequency of crises and blood transfusions did not affect the mean TAMMV values or the prevalence of a high risk of stroke. This finding agrees with previous studies that reported no association between disease severity and the presence of elevated TAMMV [12] or the risk of stroke [30]. This is not surprising, as adequate care during each episode of crisis could prevent further complications such as increased blood flow velocity. Part of the care given to SCA patients is blood transfusion, and the availability of blood when the need for transfusion arises helps to prevent severe complications such as stroke. Additionally, in this study, hydroxyurea usage had no significant effect on TAMMV or the prevalence of a high risk for stroke. This could be because only a few study participants were on the drug for a shorter duration. In a study where a significant effect was observed, the study participants were on hydroxyurea for an average of 30 months. [18] Additionally, the fact that hydroxyurea is offered to SCA patients with worsening disease severity or when complications arise will make its effects less pronounced with a shorter duration of usage. This is highlighted in Figure 10, which shows that as the risk of stroke increases, the use of hydroxyurea increases.

Limitations

The limitation of this study stems from the fact that only one of the inflammatory cells (WBCs) was assayed, without platelet or reticulocyte counts, and their inclusion could have provided more insights into the effects of inflammatory cells on SCD morbidity. Additionally, the small number of study participants on hydroxyurea with a shorter duration of usage and the use of the alkaline denaturation method for the HbF assay might have affected the results of this study. The strengths of this study stem from the correlation of clinical and laboratory parameters with both TAMMV and risks of stroke, the identification of parameters associated with risks of stroke, and drawing inferences to guide the initiation of stroke preventive measures.

## Conclusions

This study demonstrated that age, height, PCV, WBC count, and stunting status are significantly positively or negatively correlated with TAMMV and the risk of stroke. Therefore, children who have these abnormal laboratory parameters and are malnourished should be given high priority in routine TCD examinations in the setting of limited resources. Additionally, for children between the ages of two and five years who are stunted with high WBC counts and low PCV, TCD examination should be mandatory; if this examination facility is not available, stroke preventive measures should be activated. This proactive measure will facilitate early detection and management of the risk of stroke in SCA patients, especially in resource-limited countries.
